# Differential acoustic habitat use in delphinids along the Florida Atlantic coast

**DOI:** 10.7717/peerj.21547

**Published:** 2026-07-31

**Authors:** Jessica Carvalho, Greg O’Corry-Crowe, Laurent M. Chérubin

**Affiliations:** Harbor Branch Oceanographic Institute, Florida Atlantic University, Fort Pierce, FL, United States of America

**Keywords:** Habitat partitioning, Dolphin vocalization, Generalized additive models, Habitat modeling, Soniferous fish

## Abstract

Acoustic signaling is fundamental to dolphin ecology, with echolocation primarily supporting foraging, navigation, and predator avoidance, and whistles facilitating social communication. Here we investigate potential acoustic habitat differentiation in oceanic dolphins (delphinids) along the Florida Atlantic coast by identifying distinct environmental conditions associated with each signal type and investigating their relationships with soniferous fish presence. Delphinid acoustic signals were recorded during a two-month deployment of a wave glider equipped with a Remora-ST acoustic recorder. Additionally, environmental variables including sound pressure level (SPL), anthropogenic noise, soniferous fish presence, location, temperature, current speed and direction, depth, distance to the coast, salinity, and chlorophyll-a concentration were recorded. Generalized additive models (GAMs) were used to identify key predictors of whistle and echolocation occurrence, and best fit models were then used to generate predictive maps of suitable habitat for each signal type and for soniferous fish. Our results revealed unique environmental predictors of delphinid presence, depending on acoustic signal type (echolocation *vs* whistles) and distinctly different areas of high detection, which was further reflected in predicted habitat suitability. Furthermore, the presence of soniferous fish is shown to influence acoustic vocalization type and detection, offering an initial look into potential predator-prey interactions shaping delphinid vocalization behavior. This research offers valuable insight for management, particularly in identifying key foraging grounds and social hotspots and further validates the capabilities of passive acoustic monitoring *via* wave gliders for ecological insights of cetaceans.

## Introduction

The production, transmission, and reception of sounds are key components of marine ecosystems, serving as a critical tool for the survival and communication of many marine organisms. Among these, delphinids, the taxon comprising oceanic dolphins, are particularly notable for their extensive use of acoustic signals, including whistles, echolocation clicks, and buzzes, each with unique acoustic characteristics and which serve distinct biological functions (Au & Nachtigall, 1997; Herzing, 2014; Jones et al., 2020; Luís et al., 2021). Whistles are tonal frequency-modulated sounds primarily used for social interactions and individual identification (Tyack, 2000; Datta & Sturtivant, 2002; Petrella et al., 2012; Sayigh et al., 2022), while echolocation clicks are short, broadband, high-frequency pulses essential for navigation and prey detection (Au & Nachtigall, 1997; Au, 2018). Buzzes, a rapid series of clicks, occur during prey capture attempts, providing fine-scale echolocation information (Ridgway et al., 2015; Martin et al., 2019).

Environmental conditions, physical habitat features, and the surrounding soundscape influence the propagation and effectiveness of these acoustic signals (Hamilton, 1976; Kuperman, 1998; Miksis-Olds, Vernon & Heaney, 2015). The short high-frequency nature of echolocation clicks causes them to attenuate over short distances underwater (Quintana-Rizzo, Mann & Wells, 2006). However, their broadband features make them an effective tool for navigation and prey detection, especially in turbid or low visibility conditions (Harley, Putman & Roitblat, 2003; Au, 2018). In contrast, the frequency range of whistles allow them to propagate over greater distances (Quintana-Rizzo, Mann & Wells, 2006), making them ideal for maintaining group cohesion and mediating social interactions (Tyack, 1997; Jensen et al., 2012; Mustun et al., 2024).

Beyond the intrinsic characteristics of the signals themselves, the broader acoustic environment, including biological and anthropogenic sounds, affects delphinid behavior and may influence patterns of distribution. Biological sounds such as those produced by soniferous fish may provide important foraging cues that influence predator–prey dynamics (Barros, 1993; Barros & Wells, 1998; Gannon & Waples, 2006). Conversely, anthropogenic noise from vessel traffic and industrial activities can cause acoustic masking, interfering with communication, orientation, and prey detection (Buckstaff, 2004; Weilgart, 2007; Heiler et al., 2016; Erbe et al., 2019). Cetaceans have been shown to change surfacing, diving, and heading patterns, as well as acoustic signal use, in response to anthropogenic noise (Nowacek et al., 2007; Martin et al., 2023). In Brazil, Guiana dolphins have been shown to change whistle duration and rate depending on boat noise levels, indicating that vessel activity can modify social and foraging behavior (Bittencourt et al., 2017b; Marcondes, Domit & Cantor, 2025). Such behavioral changes may reduce foraging efficiency and communication space, potentially leading individuals to alter patterns of habitat use, highlighting the importance of considering soundscape components when evaluating delphinid habitat use.

In addition to the soundscape, interspecific competition for resources in overlapping ranges can influence dolphin behavior. To mitigate this competition, species—and even populations within the same species—exhibit habitat partitioning strategies including spatial and resource partitioning (Giménez et al., 2017). Spatial habitat partitioning has been observed in sympatric dolphin species along the California coast (Bearzi, 2009), in Australia (Ansmann et al., 2015) and within coastal lagoons in the Indian Ocean (Gross et al., 2009). Within Florida’s Indian River Lagoon, individual bottlenose dolphins (*Tursiops truncatus*) exhibit distinct home ranges, potentially reflecting intraspecific spatial habitat partitioning (Mazzoil et al., 2011; Durden et al., 2019; Hartel, Noke Durden & O’Corry-Crowe, 2020; Mazzoil et al., 2020). Additionally, resource partitioning has been documented among sympatric dolphin species through differential prey selection (Teixeira et al., 2021; Parra et al., 2022).

While spatial and resource partitioning among sympatric delphinid species has been well-documented, the role of acoustic habitat differentiation remains largely unexplored. Given that soundscapes significantly influence delphinid presence along the Florida Atlantic coast (Carvalho, Chérubin & O’Corry-Crowe, 2025), it is essential to investigate whether different species or populations adjust their acoustic behavior to exploit different acoustic niches. Preliminary research suggests that delphinid vocalization patterns and signal use change in response to soundscape characteristics, particularly anthropogenic noise from vessels, with dolphins prioritizing echolocation and simplifying whistles in high vessel presence (Fouda et al., 2018; Morales-Rincon et al., 2025). Understanding how delphinids utilize acoustic signals differently across habitats can provide critical insights into their ecology and inform conservation strategies.

Advances in passive acoustic monitoring, particularly the use of autonomous platforms such as wave gliders, have greatly expanded our capacity to study cetacean vocal behavior and habitat use over large spatial and temporal scales (Mellinger et al., 2007; Baumgartner et al., 2013; Klinck et al., 2016; Bittencourt et al., 2017a; Bittencourt et al., 2018; Malinka et al., 2018; Aniceto et al., 2020; Cauchy et al., 2023; Carvalho, Chérubin & O’Corry-Crowe, 2025). These autonomous platforms can operate continuously over extended periods and across vast distances, independent of weather conditions or crew availability. This capability is particularly beneficial for continuous monitoring, including during periods when visual surveys are less effective. Additionally, when equipped with environmental sensors, these platforms also allow researchers to examine the relationships between acoustic activity and oceanographic or biological variables, further enhancing our understanding of environmental drivers of delphinid presence and behavior (Chavez-Rosales et al., 2019; Carvalho, Chérubin & O’Corry-Crowe, 2025).

In this study, we utilize acoustic data from a two-month wave glider deployment along the East Florida Shelf from the start of March to the end of April, a seasonal transition period that may influence species distributions and acoustic activity, to examine: (a) habitat use among delphinid species as inferred from echolocation and whistle activity, and (b) spatial and temporal patterns of fish sound production. We apply generalized additive models (GAMs) to assess the influence of environmental and acoustic variables, including the sound production patterns of sonifying fish species, on the presence of these two signal types. Our findings reveal distinct spatial and environmental patterns for echolocation and whistle production, offering new insights into acoustic niche differentiation and habitat selection among delphinids. These results contribute to a deeper understanding of dolphin ecology and offer practical implications for acoustic-based conservation strategies.

## Methods

### Data collection

**Figure 1:**
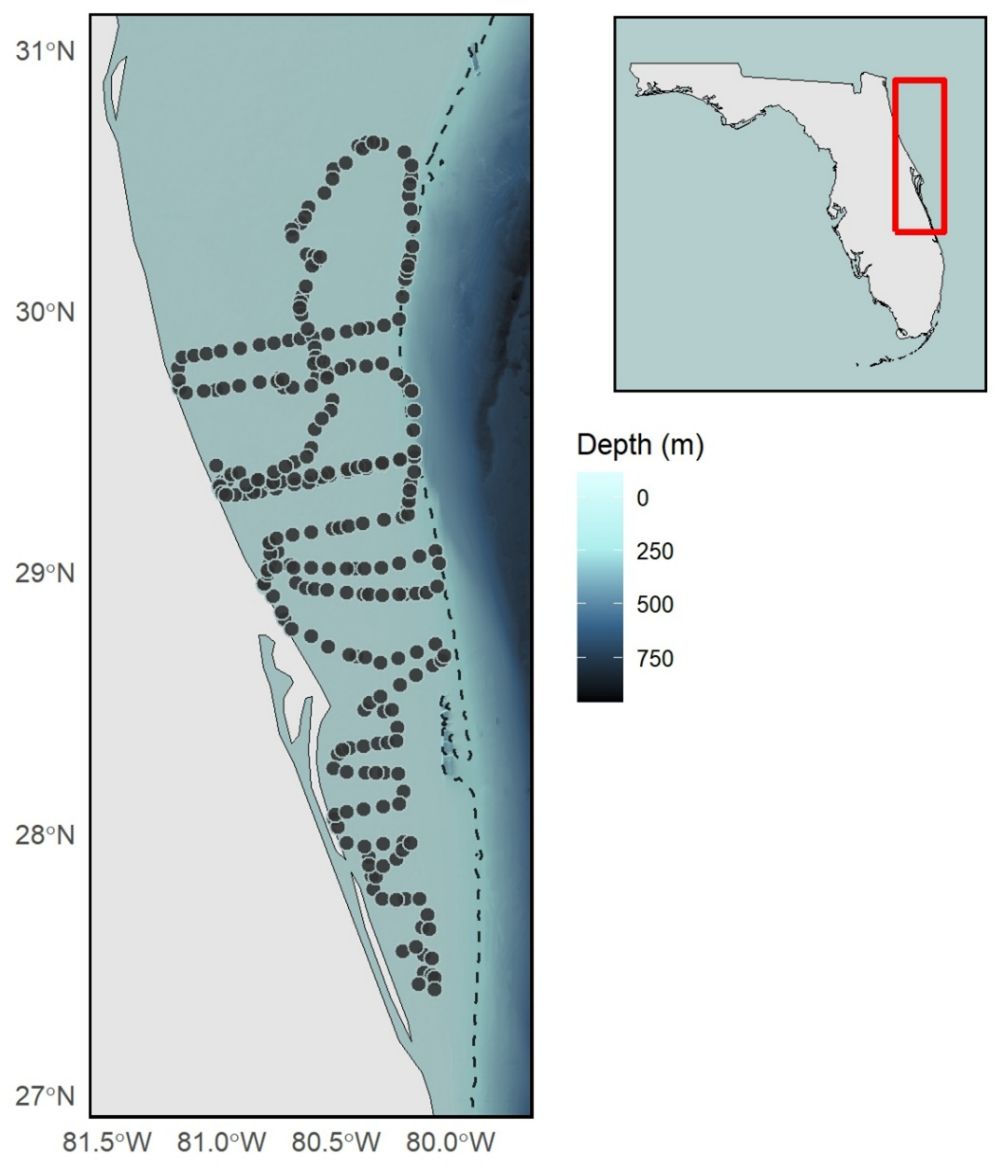
Map displaying glider track where recordings where made along the Florida Atlantic seaboard. Black dots represent the glider track and the dashed line represents the 200 m isobath.

Data for this study was collected during a two-month deployment of a Liquid Robotics autonomous wave glider along the Atlantic seaboard of Florida in March and April 2019. The survey took place within the continental shelf between Fort Pierce, Florida to the south and Jacksonville, Florida to the north (Fig. 1). The survey consisted of cross-shelf transects from the coast to the edge of the Gulf Stream. No field permits were obtained for this study as there is no field permit required to deploy an unmanned autonomous platform at sea and all acoustic recordings were made underwater, with no human subjects in the recordings. The wave glider is a wave powered autonomous surface platform, which consists of a surface float connected to a submersible glider 4 m below. The latter is connected *via* a 20 m cable to a towfish that carries the sensors payload. The towfish is ballasted to be neutrally buoyant between 4 and 10 m deep. For this survey, the towfish was equipped with an array of environmental sensors that measured pressure, temperature, salinity, dissolved oxygen, colored dissolved organic matter (CDOM), chlorophyll-a (Chl-a), and backscattering fluorescence. The surface float measured surface current and housed an acoustic doppler current profiler that measured water column current profiles down to 50 m. For further details on the glider features, instruments and payload refer to Chérubin et al. (2020) and Carvalho, Chérubin & O’Corry-Crowe (2025).

Acoustic recordings were obtained using a Remora-ST from Loggerhead Instruments mounted on the towfish. Recordings followed a duty cycle of fifteen seconds every five minutes and were made over a 54-day period between March 8th and April 30th, 2019. The Remora-ST recorded at a sampling rate of 96 kHz. Additionally, a low frequency passive acoustic monitoring system integrated into the glider payload management system sampled at a rate of 10 kHz, which was used for the assessment of soniferous fish presence along the glider track.

### Data processing

Acoustic files from the Remora-ST were reviewed using spectrograms generated in Raven Pro 1.6 with a 1,024-point DFT and 60% overlap. A single observer (JC) visually reviewed the recordings in their entirety. Signals of interest were first identified visually, followed with aural confirmation for robust validation (JC). Due to the overlap of signals throughout files, signal detection was simply classified as either present (1) or absent (0) within each fifteen-second file. These detections were then categorized as either echolocation (including buzzes) or whistles (Fig. S1). Echolocation was defined as high-frequency, broadband click trains, occasionally including rapid buzzes, though these were rarely observed. Whistles were identified as narrow-band frequency modulated signals.

Detection ranges of delphinid acoustic signals can vary by signal type and habitat characteristics (Quintana-Rizzo, Mann & Wells, 2006). Whistles may be detectable over distances exceeding 20 km, while echolocation clicks typically attenuate at shorter distances and are not typically detected at ranges greater than 1 km (Quintana-Rizzo, Mann & Wells, 2006). To address the variability of signal detection, data were aggregated at two spatial resolutions using hexagonal grids: 1 km for echolocation and 10 km for whistles. This approach helps to address the possibility of detections being produced by individuals at greater distances away from the glider’s exact location, while also providing balance in maintaining the integrity of environmental variables (see habitat variables section below), which can fluctuate on much smaller scales. Each glider observation which occurred within a grid cell was assigned that corresponding grid identification number (ID).

To address occasional irregularities in the glider’s deployment path, which resulted in disproportionately longer sampling periods in some areas, data were aggregated by both grid cell ID and date prior to statistical analysis. Specifically, all glider observations occurring within the same grid cell on the same date were averaged, producing a single daily value for each grid cell-date combination. This spatio-temporal approach allowed for better normalization of the data and addressed sampling biases due to path overlaps. All spatial analyses were performed in QGIS (Version 3.36).

### Habitat variables

Environmental variables collected by the glider sensors included salinity (ppt), subsurface temperature (°C), surface current speed (m/s), and surface current heading (degrees from the north), and Chl-a concentration (raw fluorescence units (RFU)). Additionally, physical variables including water depth (m) and distance to the coast (km) were included. Water depth was obtained from Gebco (GEBCO, 2023) and natural log transformed prior to modeling. Distance to the coast was calculated in QGIS as the shortest measured distance from the coastline to the centroid of each hexagonal grid cell.

To evaluate the influence of the soundscape on delphinid presence, additional acoustic variables were included: sound pressure level (SPL, dB re 1 µPa), and presence/absence of soniferous fish and anthropogenic noise. Time-averaged RMS sound pressure levels (SPL; dB re 1 µPa) were calculated for each file within the 300–5,000 Hz frequency band using PAMGuide (Merchant et al., 2015) in MATLAB (MathWorks 2022). These were computed using 1-s Hanning windows with 50% overlap and calibrated using the hydrophone sensitivity. The 300–5,000 Hz frequency band was chosen to capture the bulk of anthropogenic noise while minimizing interference from glider self-noise below 300 Hz. Anthropogenic noise and soniferous fish in the 0-1000 Hz frequency band were visually and aurally identified (JC) and categorized as present (1) or absent (0) in the same way as delphinid signal detections were quantified. This frequency band was selected for fish call detection because most soniferous prey species within dolphin diets (*i.e.,* scieanids, batrachoidids, sparids, haemulids, carangids, clupeids, ariids, triglids) produce sound within that frequency range (Barros, 1993; Gannon & Waples, 2006). Fish sounds were identified to the species level whenever possible and included scieanids, batrachoidids, and carangids, which were the most commonly found in the recordings (Figs. S2A–S2C). Likewise, anthropogenic noise sources were identified whenever possible, often including vessel traffic (Fig. S2D). Other biological sounds that were not identified and occurred rarely were not considered.

### Statistical analysis

Generalized additive models (GAMs) with a Tweedie distribution were used to assess the influence of environmental and acoustic variables on delphinid signal presence. The Tweedie distribution was selected for the GAM models based on its demonstrated efficacy in handling sparsely distributed species (Miller et al., 2013). The response variables in our models (echolocation detections and whistle detections) were continuous non-integer values representing spatiotemporal averages of binary (0/1) detections, making Tweedie well-suited to handle the dataset. Separate models were constructed for whistles (W10) and echolocation (E1) using the 10 km and 1 km grid scales, respectively, with each grid cell being assigned an ID number. Both initial models included all of the following variables: location (date, grid cell ID), anthropogenic noise, SPL, soniferous fish presence, distance to the coast, salinity, temperature, water depth, surface current speed and heading, and Chl-a concentration (Table 1). Furthermore, to address irregularities in the gliders path and more time spent in some areas than others, glider effort was considered in the GAMs as well.

**Table 1:** All predictor variables selected for GAM analysis of cetacean presence. Abbreviations are included for every variable, along with a description of the variable measured.

**Abbreviation**	**Description**
Ant	Anthropogenic noise (presence/absence)
SPL	Sound Pressure Level 300 Hz–5,000 Hz (dB re 1 μ Pa)
Fish	Fish (presence/absence)
Dist	Distance to the coast (km)
Sal	Salinity (ppt)
Temp	Sub surface temperature (°C)
Dep	Bathymetry (m)
CurS	Current speed (m/s)
CurH	Current heading (Degrees from the north)
Chla	Concentration of chlorophyll-a (RFU (raw fluorescence units))

After constructing the initial models, the dredge function in R Studio’s MuMIn package was used to identify an initial subset of models based on Aikake information criterion (AIC) scores. Then to further improve model fit and avoid overfitting and reduce collinearity among environmental variables, top ranked models were further evaluated based on smooth significance, concurvity diagnostics, and ecological interpretability. The best fit model to meet these parameters was selected for each signal type (Table 2).

**Table 2:** Results of top performing GAMs for E1, W10, ENF, WNF, and F1 models. Results of top performing GAMs for 1 km echolocation detections (E1 model) and 10 km whistle detections (W10 model) with fish presence included and with fish presence eliminated from the model (ENF model; WNF model) and also the top performing model for fish presence (F1). Variables included in the model are represented with (+). Aikaike information criterion (AIC) scores are included for model comparison, along with R-squared values (R-sq) and percentage of deviance explained (Dev).

	Fish	SPL	Ant	Location	Dist	Sal	Temp	Dep	CurS	CurH	Chla	AIC	R-sq	Dev
EG1	+	+		+		+		+				1,175.50	0.120	25.4%
WG10		+		+	+		+				+	−366.388	0.557	47.6%
		SPL	Ant	Location	Dist	Sal	Temp	Dep	CurS	CurH	Chla	AIC	R-sq	Dev
ENF		+		+			+	+		+		1,176.754	0.122	26.5%
WNF		+		+	+		+				+	−366.388	0.557	47.6%
		SPL	Ant	Location	Dist	Sal	Temp	Dep	CurS	CurH	Chla	AIC	R-sq	Dev
F1		+		+		+			+		+	2,043.769	0.363	36.2%

This process was also repeated for two additional models, in which fish presence was removed from the initial whistles (WNF) and echolocation (ENF) models. This allowed us to better assess the influence of fish presence on dolphin presence and to understand the contribution of fish presence to the models (Table 2).

### Model adequacy

Model adequacy for the best fit model E1 and W10 model was conducted using standard diagnostic procedures for GAMs. The gam.check() function from the mgcv package in R was used to assess model convergence, basis dimension adequacy and distributional assumptions through residual analysis. Basis dimension sufficiency was evaluated using k-index diagnostics. Additional diagnostic plots, including QQ plots, residuals *versus* linear predictor plots, histograms of residuals, and observed *versus* fitted value plots, were used to assess potential model misspecification and overall fit. Corresponding k-index diagnostics and visual diagnostic figures are provided in the Supplementary Material (Table S1; Figs. S3–S4).

### Predictive modeling

Once the top model was identified for both echolocation and whistles, the selected models were used for predictive modeling. These predictive models generated a habitat suitability estimate for each location along the glider path and were used to create maps to visualize potential suitable delphinid habitats in two separate behavioral states along the glider track.

To further explore the relationship between delphinid activity and fish habitat suitability, GAMs were also run with fish presence as the response variable, using the same set of predictors (Table 2). These models were built at the 1 km scale due to the limited detection range of fish calls. The top resulting model was then used to run a predictive model of habitat suitability for soniferous fish.

## Results

Nearly 62 h of acoustic recording were reviewed throughout this study, accounting for a total of 14,974 recorded files. Dolphins were detected in just over 11% of the recordings (*n* = 1,691 files). Whistles were the most commonly detected dolphin signal, accounting for 55.4% of detections, echolocation signals trailed behind at 29.5%, and the remaining 15.1% contained a combination of both whistles and echolocation signals.

### Whistles

**Figure 2:**
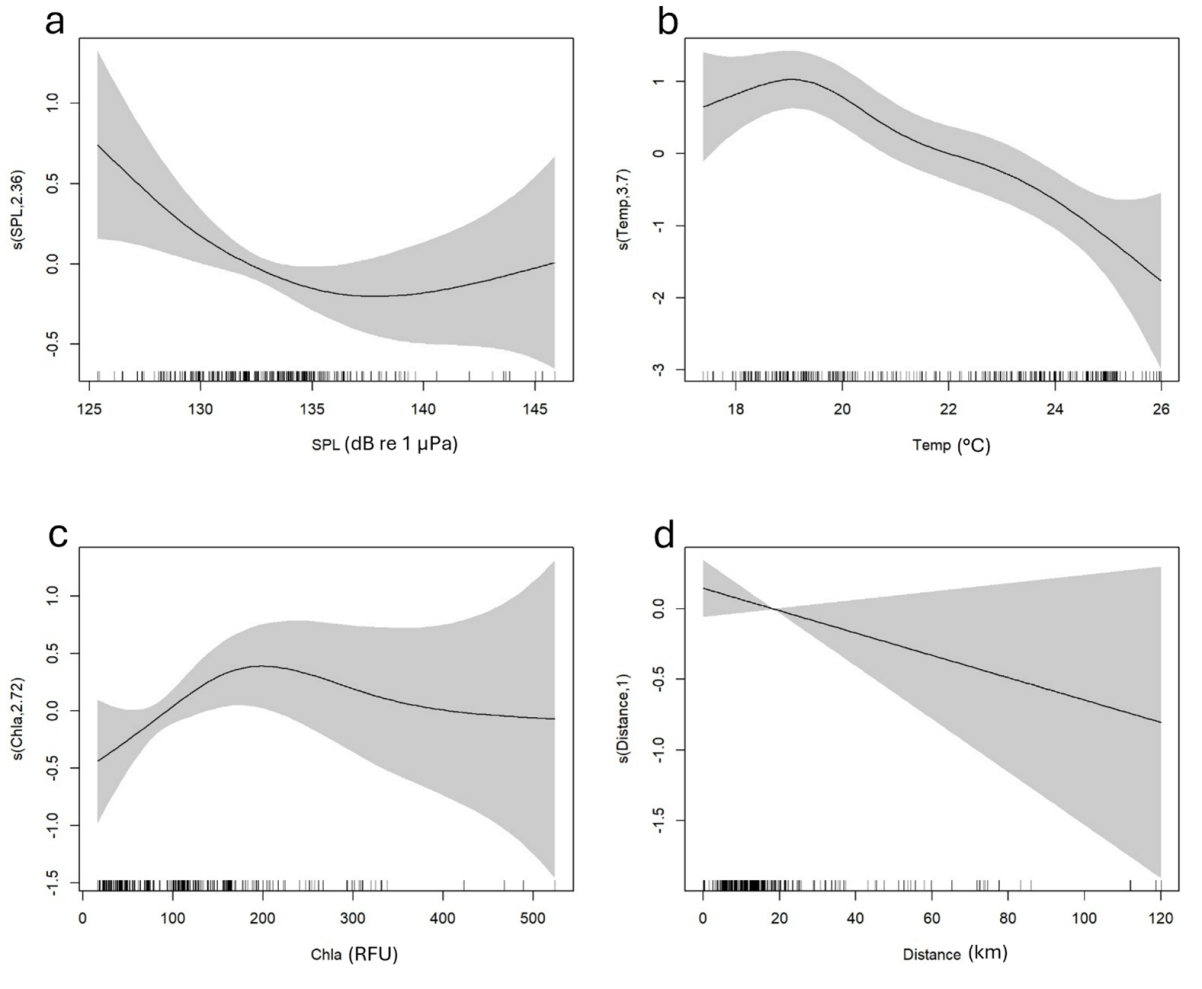
Smooth plots showing results of the top performing W10 Model. (A) SPL (dB), (B) sub surface sea temperature (°C), (C) chl-a concentration (RFU), (D) distance to the coast (km). Tick marks on the *x*-axis represent data observations and gray shading represents the 95% confidence interval. The number associated with each variable represents the effective degrees of freedom (EDF) for each smooth term, indicating the complexity of each variable’s relationship with cetacean presence, 1 being the least complex.

**Figure 3:**
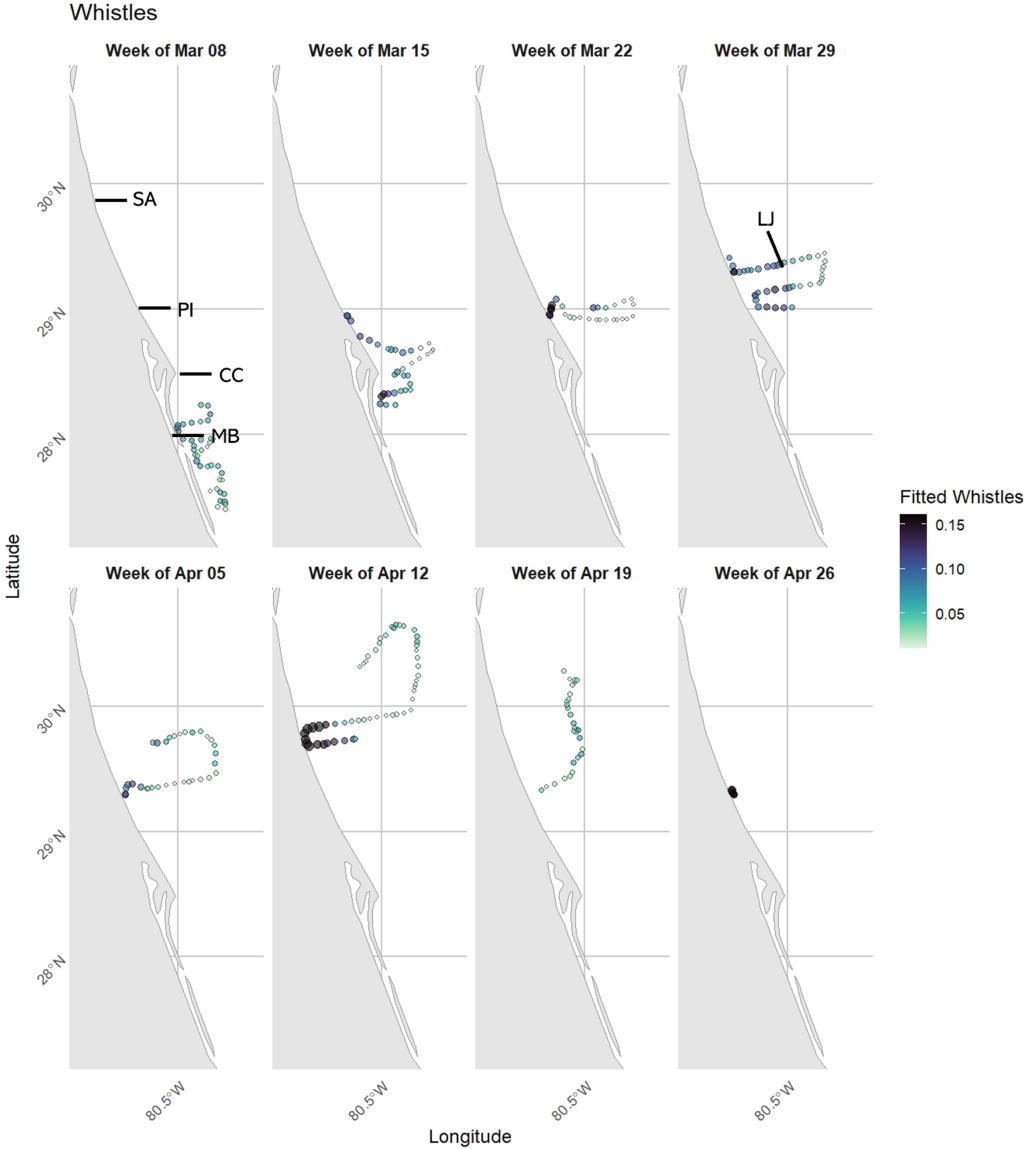
GAM results of the influence of location (date/ID) on weekly presence of whistle detections along the glider track. Each map represents one week of the glider deployment. Darker colors and larger points represent areas of more whistle detections. Labels mark areas of interest: St Augustine (SA), Ponce Inlet (PI), Cape Canaveral (CC), Melbourne Beach (MB), Long John reef (LJ).

The top W10 model explained 47.6% of the deviance in the data set with an R squared of 0.557 and an AIC score of −366.388 (Table 2). Model diagnostics indicated adequate fit, with simulated QQ plots showing good agreement with expected distributions and residuals displaying no strong patterns with the linear predictor (Fig. S3). The top model included sound pressure level (SPL), location (date, ID), temperature, distance to the coast, and Chl-a concentration. Among these variables, SPL, location, temperature, and Chl-a were statistically significant, whereas distance to the coast was not which are represented by *p*-values in Table S2. SPL generally exhibited a negative correlation with whistle presence, though a slight positive trend was observed at higher SPL values (Fig. 2A). Temperature had a weak positive correlation at cooler temperatures and displayed a clear negative correlation with whistle presence at warmer temperatures (Fig. 2B). Whistle presence was negatively associated with distance to the coast (Fig. 2D) and had a complex relationship with Chl-a concentration (Fig. 2C), with increasing whistle detections up to 200 RFU, but a slight negative relationship thereafter. Whistle detections showed a generally positive relationship with locations closer to shore and showed areas of increased modeled whistle activity near St. Augustine, Ponce Inlet, north of Melbourne beach, and along the coast just north of Ponce Inlet (Fig. 3).

### Echolocation

**Figure 4:**
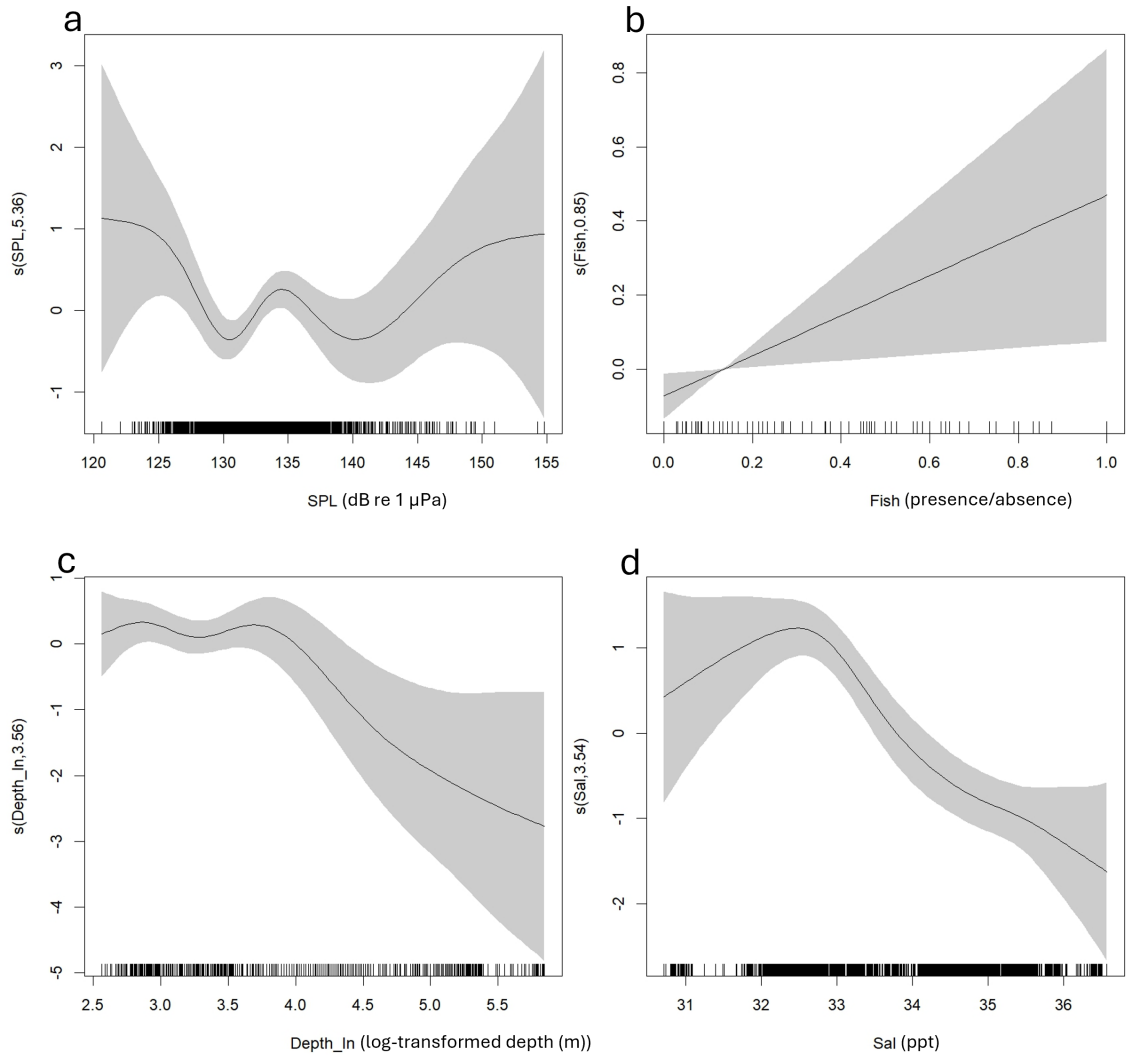
Smooth plots showing results of the top performing E1 Model. (A) SPL (dB), (B) fish presence (presence/absence), (C) depth (log-transformed (m)), and (D) salinity (ppt). Tick marks on the *x*-axis represent data observations and gray shading represents the 95% confidence interval. The number associated with each variable represents the effective degrees of freedom (EDF) for each smooth term, indicating the complexity of each variable’s relationship with cetacean presence, 1 being the least complex.

**Figure 5:**
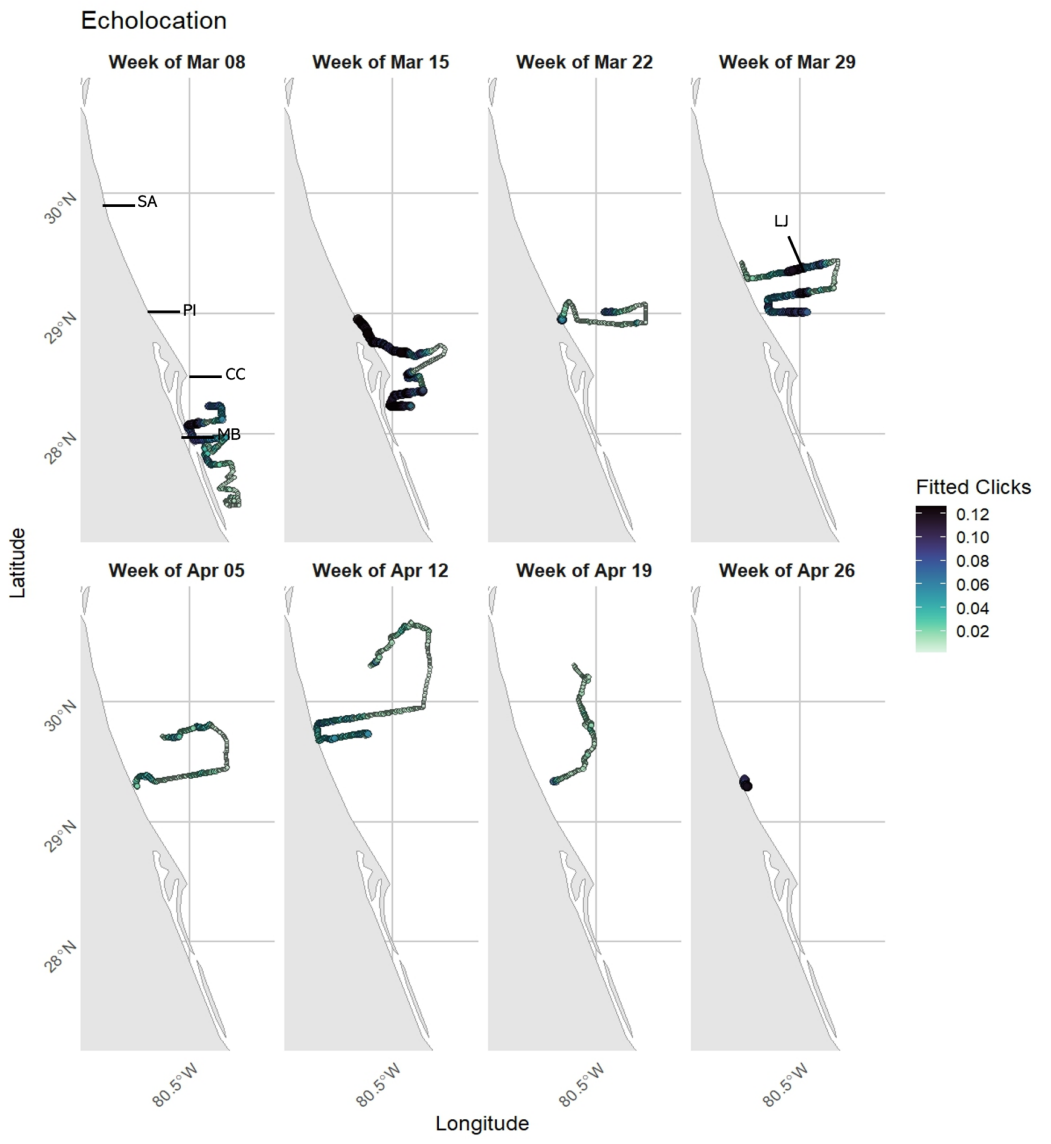
GAM results of the influence of location (date/ID) on the weekly presence of echolocation detections along the glider track. Each map represents one week of the glider deployment. Darker colors and larger points represent areas of more echolocation detections. Labels mark areas of interest: St Augustine (SA), Ponce Inlet (PI), Cape Canaveral (CC), Melbourne Beach (MB), Long John reef (LJ).

The top E1 model explained 25.4% of deviance in the dataset with an R-squared of 0.120 and an AIC score of 1,175.50 (Table 2). Model Diagnostic plots suggested an overall adequate fit, with simulated QQ plots broadly following the expected distribution (Fig. S4). The top model included SPL, fish presence, location, salinity, and depth. All variables within this model were statistically significant, which is shown by the *p*-values in Table S2. The resulting smooth plots for all variables in the top E1 model can be seen in Figs. 4 and 5. SPL had a complex relationship with echolocation presence, with peaks at 125, 135 and 155 dB (Fig. 4A). Delphinid echolocation was positively associated with fish presence (Fig. 4B). Depth had a predominantly negative association with echolocation detections, with detections declining in deeper waters (Fig. 4C). Echolocation peaked with salinity around 32.5 ppt (Fig. 4D) and declined thereafter. Echolocation detections were higher south of Cape Canaveral and Ponce Inlet, but there was also a notable area of increased modeled echolocation activity offshore, seemingly near Long John reef; a hard bottom habitat also targeted by fishermen (Fig. 5). Another small area of increased activity could be identified between St. Augustine and Ponce Inlet.

### Fish

The top ENF model explained 26.5% of deviance in the dataset with an R-squared of 0.122 and an AIC score of 1,176.754 (Table 2). This model included SPL, location, temperature, depth, and current heading. This model performed fractionally better than the E1 model in explained deviance but had a higher AIC score. The top WNF model was the same as the W10 model (Table 2).

### Habitat suitability

**Figure 6:**
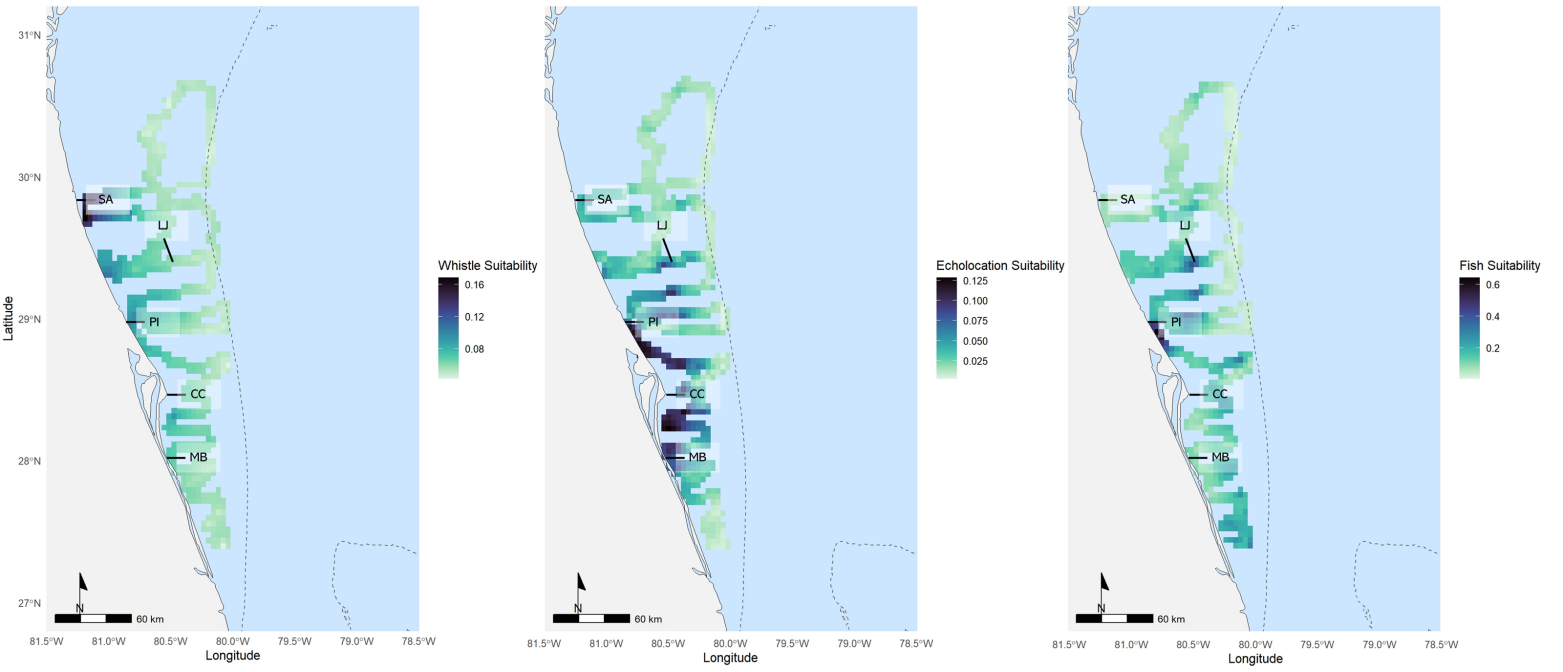
Suitable habitat maps of dolphin whistles and echolocation, and fish. Maps displaying predicted suitable habitats for whistles (A), echolocation (B), and fish (C). Darker colors represent areas of greater suitability. The dashed line represents the 200m isobath. Labels mark areas of interest: St Augustine (SA), Ponce Inlet (PI), Cape Canaveral (CC), Melbourne Beach (MB), Long John (LJ).

The predicted suitable habitat for delphinids varied between whistle and echolocation detections (Figs. 6A & 6B). The predicted suitable habitat for echolocation was concentrated around Melbourne and Cape Canaveral extending north toward Ponce Inlet, with an additional patch of suitability seen offshore, likely associated with Long John reef. In contrast, the predicted suitable habitat for whistles was strongly concentrated around St. Augustine. These spatial differences may indicate distinct ecological preference or behavioral association with habitat use.

The predicted suitable habitat for fish displayed some overlap with suitable habitat for delphinids (Fig. 6C). Fish suitable habitat coincided slightly with echolocation habitat suitability. Predicted suitable habitat for fish was highest around Ponce Inlet and offshore near Long John reef. Another area of moderate habitat suitability appeared in the southern region of the study area near a series of offshore artificial reefs such as sunken vessels and bridge construction material (St Lucie County Artificial Reef Monitoring Program; Fig. 6).

## Discussion

Our study highlights distinct patterns of habitat use associated with delphinid whistles and echolocation signals, underscoring the important role of these acoustic signals on delphinid behavior. Spatiotemporal patterns of dolphin vocalizations were highly non-uniform with clear areas of increased detection and areas lacking detections. The distribution of high detection and low detection locations was markedly different between echolocation clicks and whistles. These patterns were also reflected in modeled habitat suitability, with non-uniform patterns of suitability seen, which often reflected the original observed distributions, but strikingly, differed in some key areas.

### Whistles

Whistles, predominantly used for social communication, play a critical role in maintaining group cohesion, individual identification, and even long-range communication (Quick & Janik, 2012; Janik & Sayigh, 2013; Herzing, 2014; Janik, 2014). We found that whistle detections were influenced by environmental and spatial factors, including location, SPL, water temperature, distance to the coast, and Chl-a concentration along the Florida coast. Interestingly, the detection of soniferous fish was not an impactful variable in explaining the distribution of dolphin whistles. As previously mentioned, dolphins primarily use whistles and other frequency modulated signals during social interactions with conspecifics which may not always coincide with foraging behavior and thus fish presence. Conversely, soniferous fish may react to dolphin whistles (which propagate over great distances) by ceasing to produce sounds as a predator avoidance strategy (Remage-Healey, Nowacek & Bass, 2006; Ladich, 2022), which could further contribute to fish calls not being a good predictor of dolphin whistle activity.

Whistle detections were more frequent nearshore, with two prominent modeled whistle activity areas clearly identified near St. Augustine and Ponce Inlet and to a lesser extent just north of Melbourne Beach and near shore north of Ponce Inlet. Interestingly, though included in the top model, distance to the coast was not statistically significant, suggesting a more complex relationship to habitat selection than a simple proximity to the shoreline. The observed increased activity around St. Augustine was reflected heavily in the predicted suitable habitat model. However, the observed increased activity near Ponce Inlet and just north of it, were only lightly reflected in the habitat suitability model, and the small area around Melbourne Beach was not reflected much in this model. The highlighted areas of high habitat suitability experience high boat traffic, which can produce mixed results in whistle detections. Increased noise has been shown to mask dolphin signals (Jensen et al., 2009; Erbe et al., 2016), which can impact our ability to detect them and may lead to missed detections, but it is not possible to quantify this within our data. However, it has also been documented that increased noise can drive increased whistle activity as dolphins maintain group cohesion and navigate through noisy, vessel-dense environments (Nowacek, Wells & Solow, 2001; La Manna et al., 2013). The slight uptick in whistle detections at higher SPL levels in our dataset may reflect behavioral trade-offs, whereby dolphins tolerate noise in areas with other benefits (*e.g.*, prey availability, oceanographic features) or may indicate that some noise originates from biological rather than anthropogenic sources (*e.g.*, wave action, snapping shrimp).

Significantly, perhaps there appears to also be a temporal pattern to whistle activity across the two-month deployment. In some weeks, for example, we had fewer detections than in others (Fig. 3). This pattern, also evident in echolocation detections, may include seasonal movements in delphinids along the coast (see below).

Environmental influences on whistles extended beyond soundscape conditions. Whistle detections were negatively associated with warmer water temperatures. Previous research has found dolphin group sizes diminish with increased water temperature and species show preference for cooler waters (Chavez-Rosales et al., 2019; Tardin et al., 2019; La Manna et al., 2023). Conversely, whistle detections increased with rising Chl-a concentration in areas where model 95% confidence was highest, which coincides with previous findings linking primary productivity with dolphin distribution (Scott et al., 2010; Tardin et al., 2019; Sahri et al., 2021). At the highest Chl-a concentrations whistle detections showed some decline and leveling off, though limited data in this range introduces uncertainty. Relatively high Chl-a is consistent with habitats such as inlets where productivity is increased in estuarine environments. Lower temperatures that were shown as a preferred environment are also characteristic of the nearshore waters with estuarine properties.

### Echolocation

In contrast to whistles, delphinid echolocation, used predominantly for navigation and prey detection (Au & Nachtigall, 1997; Branstetter & Mercado III, 2006; Au, 2018), was influenced by a different set of variables, including location, depth, SPL, fish presence, and salinity. The contrast of these variables underscores the distinction between social and foraging behaviors and their associated ecological needs. Echolocation detections were concentrated in a wide band centered around Cape Canaveral extending from Melbourne in the south to Ponce Inlet in the north and offshore, likely associated with Long John reef. Echolocation detections were highest at moderate salinity levels, potentially reflecting use of nearshore waters influenced by riverine inputs (*e.g.*, Indian River Lagoon). Shallow waters were positively associated with echolocation, whereas in deep waters, detections declined. The nuanced relationship between SPL and echolocation suggests possible masking effects (Jensen et al., 2009; Erbe et al., 2016) or behavioral response to noise where echolocation increases with noise levels (Nowacek, Wells & Solow, 2001; La Manna et al., 2013).

In contrast to fish calls not being a good predictor of dolphin whistle activity, there was a positive association between soniferous fish presence and delphinid echolocation in the top model. This suggests that dolphins may exploit acoustic cues by fish (*e.g.*, grunts, drumming) to identify foraging hotspots, consistent with prior findings of regional prey preference for soniferous species (Barros, 1993; Barros & Wells, 1998; Gannon & Waples, 2006). Areas of increased echolocation modeled activity aligned with predicted suitable habitat, particularly around Cape Canaveral and offshore near Long John reef, which is a known site of high fish presence. These areas may represent critical foraging grounds for dolphins.

As with whistle detections, there is an apparent temporal component to dolphin echolocation. There tends to be a higher number of detections, for example, in the first month (March) of the glider deployment compared to the second month (April). In this region of the Atlantic Ocean, migratory stocks of dolphins are known to overlap seasonally with resident stocks. For example, in April the southern migratory coastal stock of bottlenose dolphins begins its return journey north and vacates northern Florida’s coastal Atlantic waters (Hayes et al., 2021). These temporal patterns of whistles and echolocations may be attributed to, at least in part, seasonal movements of delphinids along the Atlantic coast. This highlights the potential of autonomous platforms to monitor complex movements of multiple species and stocks.

### Fish presence

Patterns in fish presence models revealed relatively weak associations with most environmental predictors, except for location, which emerged as the strongest driver of fish presence (Table 2). As a consequence, fish presence had a limited influence on echolocation as shown by the minimal effect on model results, when fish were removed as a predictor variable in the ENF and WNF models (Table 2). However, fish habitat suitability somewhat overlapped with echolocation suitability, with increased activity offshore in the northern area of the study region near Long John reef and around Ponce Inlet. It suggests that soniferous fish presence coincides with potential foraging behavior on these species. Furthermore, another area of suitable habitat emerged in the southern region of the study area near artificial reef sites, where there was no suitability for echolocation. This mismatch may be indicative of selective predation and predator/prey interactions. Fish could produce sound in areas where they are less vulnerable or refrain from sound production where predation pressure is high. Habitat differences between northern (natural hard bottom) areas and southern artificial reef-associated areas may also contribute to this contrast. Therefore, fish presence remains an important factor in echolocation activity despite the fact that fish may reduce sound production then, which is reflected by a reduced influence in E1.

Across our top models (W10, E1, F1), location consistently explained a large portion of the variation in the models. This result indicates a strong spatial component to habitat selection, meaning both dolphins and fish select specific habitats for multiple interacting factors rather than single environmental drivers. Dolphins in particular have a patchy distribution which is likely a result of being a highly mobile and highly social species that exploit patchy resources.

While our study provides new insights into acoustic habitat selection of delphinids along the Florida Atlantic coast, there are certain limitations that must be acknowledged. While acoustic monitoring is an excellent noninvasive tool, it may not fully capture presence of all individuals, as both dolphins and fish produce sound intermittently. Animals may be present, but silent, or their signals may be masked by noise (Mellinger et al., 2007). Furthermore, with only a single data observer it is not possible to estimate bias in the observation. It is also important to consider the influence of species-specific behavior. Multiple dolphin species exist within the study area, including coastal and offshore taxa that differ in habitat preferences, movement patterns, group composition, and acoustic behavior, each with different ecological needs and behaviors. Because visual confirmation was not available, species identification was not possible from acoustic data alone. Consequently, some of the variability observed in acoustic presence and habitat associations may reflect differences among species rather than environmental drivers alone. Future studies integrating visual surveys could help distinguish these effects. Additionally, results from this study are based on a single glider deployment, and repeated surveys would strengthen the understanding of the patterns found.

## Conclusion

In conclusion, our findings provide evidence of acoustic habitat differentiation in dolphins, with whistles and echolocation reflecting distinct ecological roles and habitat associations. Furthermore, we highlight a potential acoustic predator–prey linkage between dolphins and soniferous fish. These results not only advance understanding of delphinid habitat use but also offer valuable insight for management, particularly in identifying key foraging grounds and social hotspots where anthropogenic disturbance may be most disruptive. Future research, further investigating predator–prey dynamics and anthropogenic noise, could advance our understanding of the role of the soundscape on dolphin behavior and related habitat selection.

##  Supplemental Information

10.7717/peerj.21547/supp-1 Supplemental Information 1 Spectrograms of echolocation and whistles Examples of spectrograms of (a) echolocation clicks and (b) delphinid whistles recorded by the wave glider. Spectrograms were calculated with a FFT size of 9600 points with a Hanning window and 80% overlap, and show the relative intensity in dB.

10.7717/peerj.21547/supp-2 Supplemental Information 2 Spectrograms of fish sounds Images showing spectrograms of (a) toadfish (Batrachoididae), (b) black drums (Sciaenidae), (c) jacks (Carangidae), and (d) anthropogenic noise from a boat. Spectrograms were calculated with a FFT size of 9600 points with a Hanning window and 80% overlap, and show the relative intensity in dB.

10.7717/peerj.21547/supp-3 Supplemental Information 3 GAM diagnostic plots for W10 model showing model fit and residual behavior Top left: QQ plot of deviance residuals. Top right: residuals vs. linear predictor. Bottom left: histogram of residuals. Bottom right: observed vs. fitted values.

10.7717/peerj.21547/supp-4 Supplemental Information 4 GAM diagnostic plots for E1model, showing model fit and residual behavior Top left: QQ plot of deviance residuals. Top right: residuals vs. linear predictor. Bottom left: histogram of residuals. Bottom right: observed vs. fitted values.

10.7717/peerj.21547/supp-5 Supplemental Information 5 Diagnostic outputs from gam.check() for the E1 and W10 models For each predictor, the table shows the smoothing parameter (k), estimated degrees of freedom (edf), k-index (a check for sufficient smoothing), and p-values testing whether edf is significantly different from k.

10.7717/peerj.21547/supp-6 Supplemental Information 6 Additional statistical results for top E1, ENF, W10, and WNF models Represented are the complexity of each smooth term, estimated degrees of freedom (edf) and reference degrees of freedom (Ref.df; available degrees of freedom). Also included is the F statistic (F) representing the contribution of each term to the model, and the P-value, representing statistical significance.

10.7717/peerj.21547/supp-7 Supplemental Information 7 GAM codes All of the coding used to run generalized additive models.

10.7717/peerj.21547/supp-8 Supplemental Information 8 Daily data on a 10km scale All detection data averaged on a daily basis on a 10km spatial scale. This data was used for all analyses of whistle detections.

10.7717/peerj.21547/supp-9 Supplemental Information 9 Daily data on 1km scale All detection data averaged on a daily basis on a 1km spatial scale. This data was used for all analyses of echolocation and fish presence data.

## Additional Information and Declarations

### Data Availability

The following information was supplied regarding data availability: The code and raw data are available in the Supplemental Files.
