# Supplementary figures and images for: Differential acoustic habitat use in delphinids along the Florida Atlantic coast
Source: PeerJ. 2026 Jul 31;14:e21547. doi: 10.7717/peerj.21547 (PMC13431293; doi:10.7717/peerj.21547)

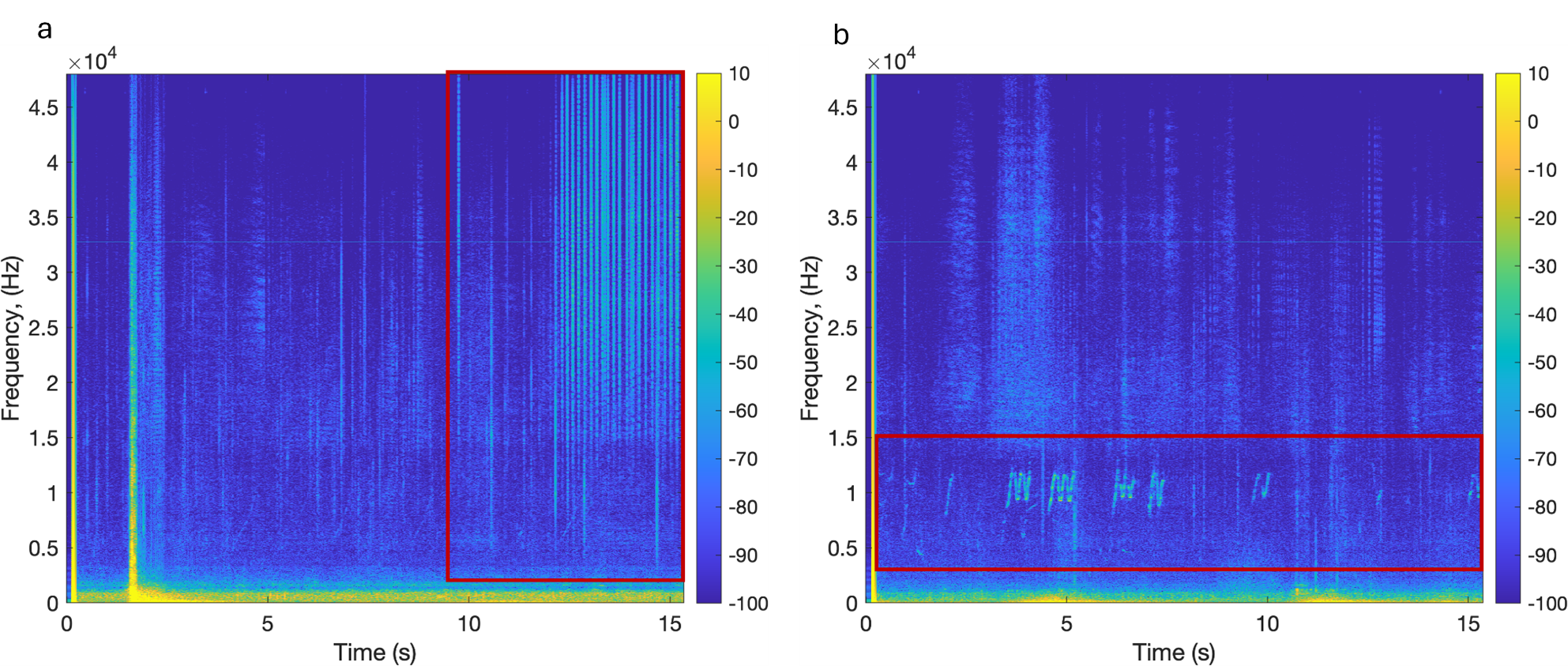

Supplement: Supplemental Information 1 — Examples of spectrograms of (a) echolocation clicks and (b) delphinid whistles recorded by the wave glider. Spectrograms were calculated with a FFT size of 9600 points with a Hanning window and 80% overlap, and show the relative intensity in dB. [file peerj-14-21547-s001.png]

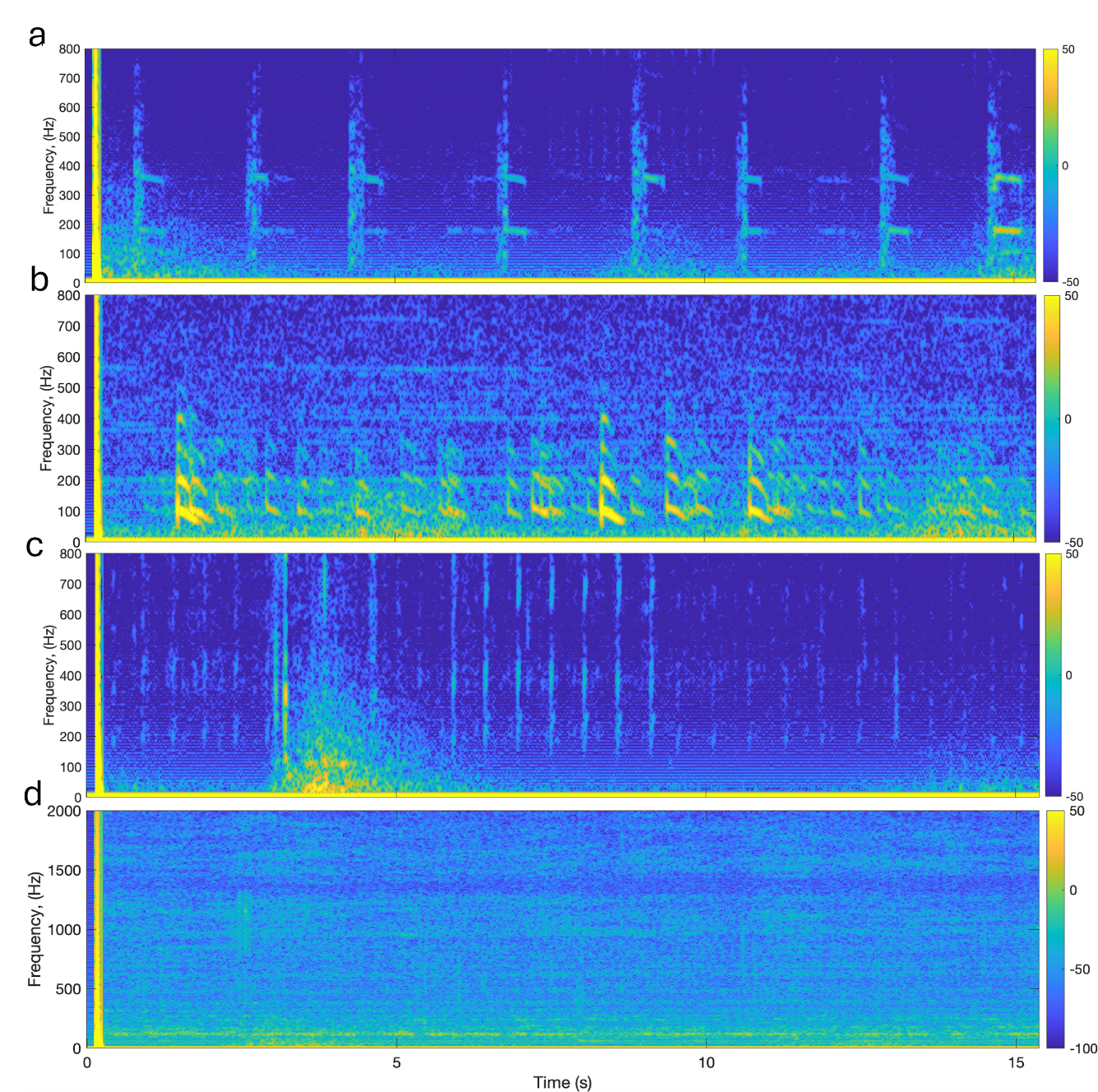

Supplement: Supplemental Information 2 — Images showing spectrograms of (a) toadfish (Batrachoididae), (b) black drums (Sciaenidae), (c) jacks (Carangidae), and (d) anthropogenic noise from a boat. Spectrograms were calculated with a FFT size of 9600 points with a Hanning window and 80% overlap, and show the relative intensity in dB. [file peerj-14-21547-s002.png]

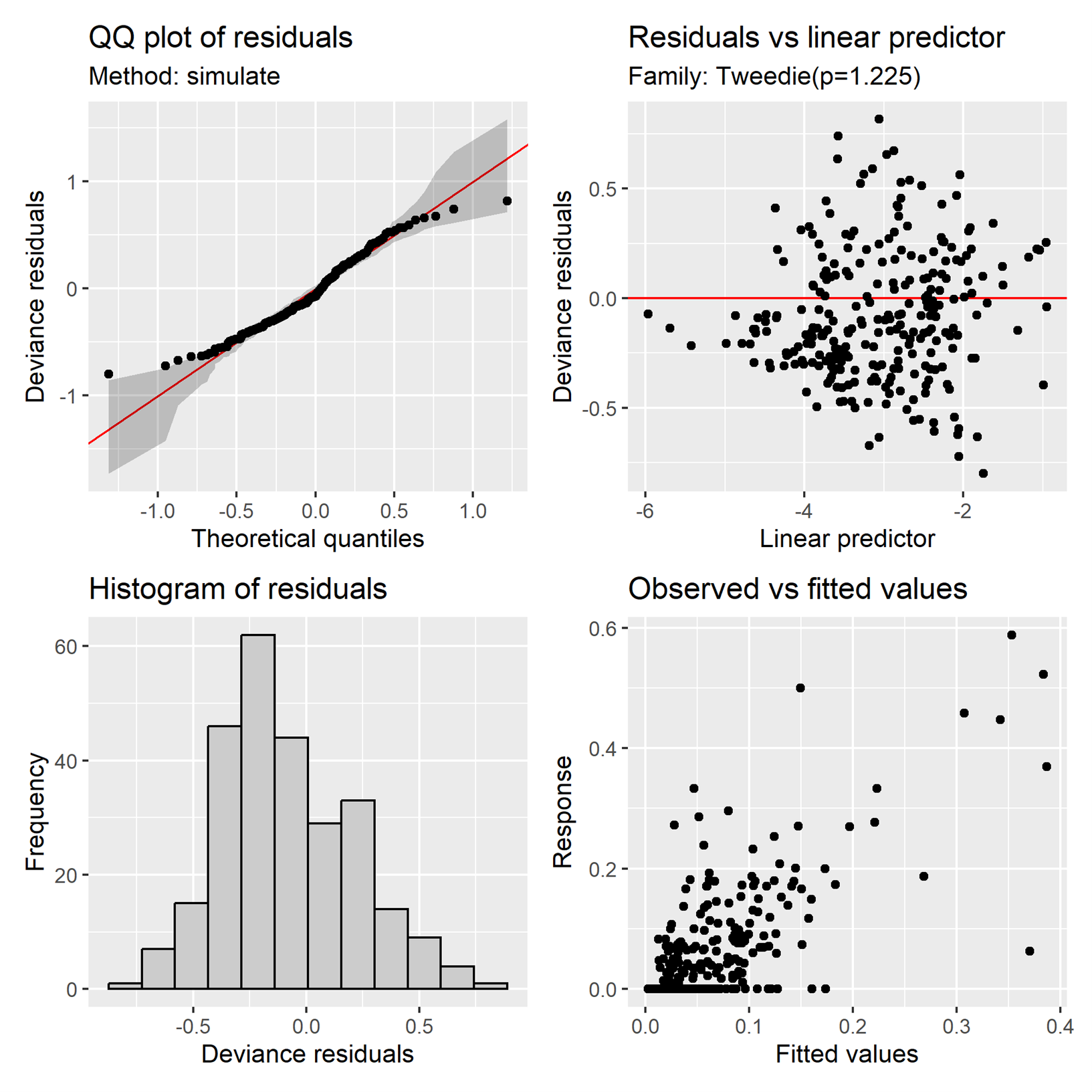

Supplement: Supplemental Information 3 — Top left: QQ plot of deviance residuals. Top right: residuals vs. linear predictor. Bottom left: histogram of residuals. Bottom right: observed vs. fitted values. [file peerj-14-21547-s003.png]

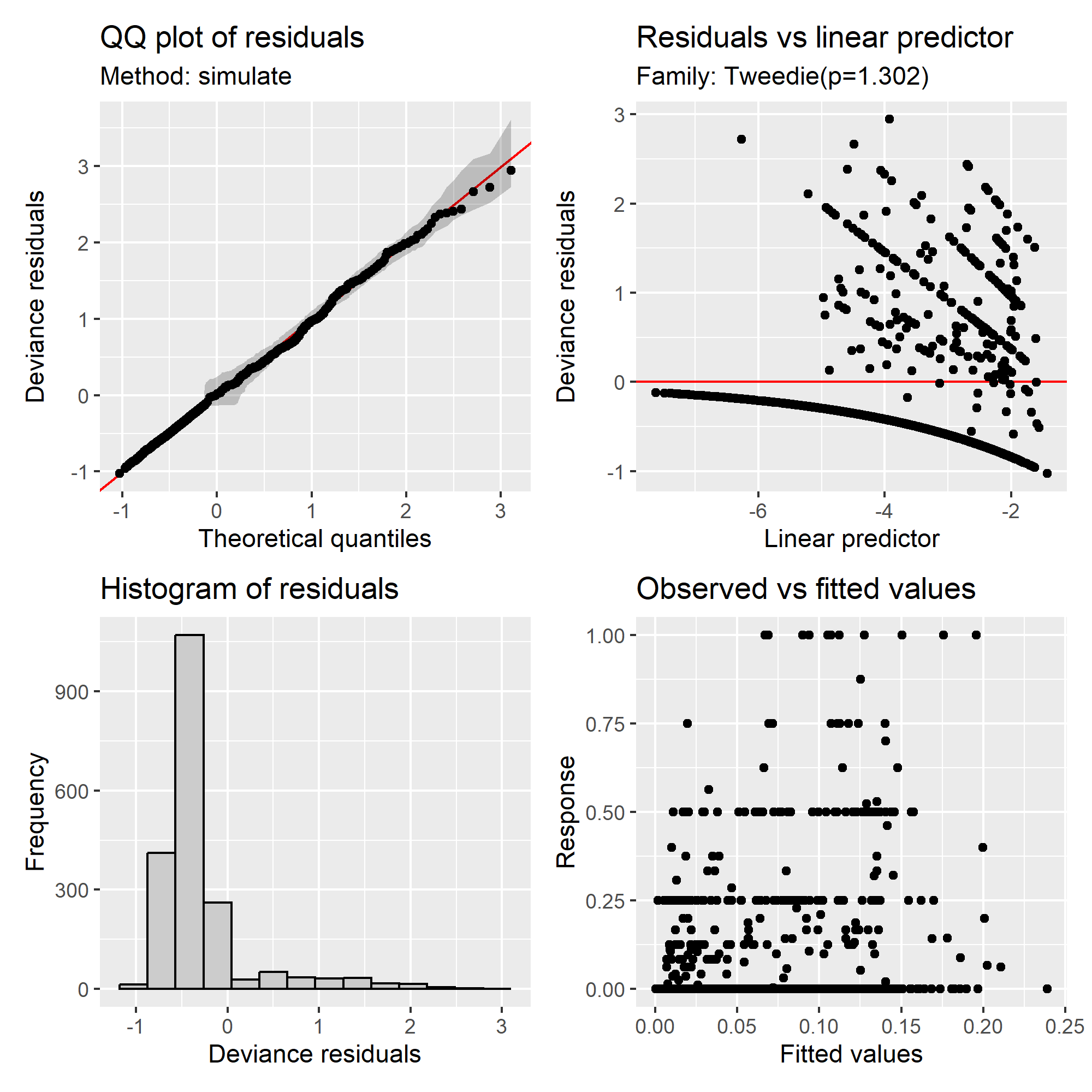

Supplement: Supplemental Information 4 — Top left: QQ plot of deviance residuals. Top right: residuals vs. linear predictor. Bottom left: histogram of residuals. Bottom right: observed vs. fitted values. [file peerj-14-21547-s004.png]
