# Supplementary material for: Differential acoustic habitat use in delphinids along the Florida Atlantic coast
Source: PeerJ. 2026 Jul 31;14:e21547. doi: 10.7717/peerj.21547 (PMC13431293; doi:10.7717/peerj.21547)
Supplement: Supplemental Information 5 — For each predictor, the table shows the smoothing parameter (k), estimated degrees of freedom (edf), k-index (a check for sufficient smoothing), and p-values testing whether edf is significantly different from k. [file peerj-14-21547-s005.docx]

| E1 Model | SPL | Location | Dep | Sal | Fish |
| --- | --- | --- | --- | --- | --- |
| k | 9.00 | 299 | 9.00 | 9.00 | 9.00 |
| edf | 5.362 | 6.791 | 3.565 | 3.537 | 0.851 |
| k-index | 0.78 | 0.72 | 0.71 | 0.79 | 0.67 |
| p-value | 0.285 | 0.005 | <2e-16 | 0.530 | <2e-16 |
| W10 Model | SPL | Location | Dist | Chla | Temp |
| k | 9.00 | 24.00 | 9.00 | 9.00 | 9.00 |
| edf | 2.61 | 8.58 | 1.00 | 3.60 | 3.55 |
| k-index | 0.93 | 0.87 | 0.98 | 0.89 | 1.01 |
| p-value | 0.48 | 0.10 | 0.84 | 0.24 | 0.95 |
