# Supplementary material for: Differential acoustic habitat use in delphinids along the Florida Atlantic coast
Source: PeerJ. 2026 Jul 31;14:e21547. doi: 10.7717/peerj.21547 (PMC13431293; doi:10.7717/peerj.21547)
Supplement: Supplemental Information 6 — Represented are the complexity of each smooth term, estimated degrees of freedom (edf) and reference degrees of freedom (Ref.df; available degrees of freedom). Also included is the F statistic (F) representing the contribution of each term to the model, and the P-value, representing statistical significance. [file peerj-14-21547-s006.docx]

| E1 Model | SPL | Location | Dep | Sal | Fish |
| --- | --- | --- | --- | --- | --- |
| edf | 5.362 | 6.791 | 3.565 | 3.537 | 0.851 |
| Ref.df | 9 | 226 | 9 | 9 | 9 |
| F | 2.156 | 0.220 | 1.669 | 6.986 | 0.630 |
| P-value | 0.00083 | <2e-16 | 0.00052 | < 2e-16 | 0.00843 |
| ENF Model | SPL | Location | Dep | CurH | Temp |
| edf | 4.923 | 11.545 | 2.662 | 0.00016 | 5.467 |
| Ref.df | 9 | 226 | 9 | 9 | 9 |
| F | 1.672 | 0.366 | 1.182 | 0.000 | 6.489 |
| P-value | 0.00421 | < 2e-16 | 0.00076 | 0.9221 | < 2e-16 |
| W10 Model | SPL | Location | Dist | Chla | Temp |
| edf | 2.614 | 8.582 | 1.002 | 3.597 | 3.546 |
| Ref.df | 3.255 | 10.393 | 1.004 | 4.457 | 4.417 |
| F | 3.724 | 5.967 | 2.564 | 2.965 | 6.441 |
| P-value | 0.0107 | < 2e-16 | 0.1109 | 0.0180 | 3.77e-05 |
| WNF Model | SPL | Location | Dist | Chla | Temp |
| edf | 2.614 | 8.582 | 1.002 | 3.597 | 3.546 |
| Ref.df | 3.255 | 10.393 | 1.004 | 4.457 | 4.417 |
| F | 3.724 | 5.967 | 2.564 | 2.965 | 6.441 |
| P-value | 0.0107 | < 2e-16 | 0.1109 | 0.0180 | 3.77e-05 |
